# Women and Their Pattern of Use of Novel Psychoactive Substances

**DOI:** 10.1192/bjo.2025.10690

**Published:** 2025-06-20

**Authors:** Win Thet, Owen Bowden-Jones

**Affiliations:** RCPsych, London, United Kingdom

## Abstract

**Aims:** This study aims to explore the patterns of psychoactive substance use among women attending the Club Drugs Clinic. It also explores associations with demographic factors and mental health comorbidities to identify additional therapeutic needs beyond current clinical treatments. The findings will help contribute to improving service provision for this population.

**Methods:** This is a quantitative study of 27 female patients who attended the Club Drugs Clinic across three boroughs between May 2021 and June 2024. Data collected includes demographic information, primary, secondary, and tertiary use of Novel Psychoactive Substances (NPS), age of onset of substance misuse, age of onset of treatment, associated mental health comorbidities, and harmful alcohol use.

**Results:** The majority of female patients attending the Club Drugs Clinic are of White British origin, with 20% identifying as Asian, Brazilian, or African Caribbean.

The average age of onset of psychoactive substance use is 25 years, while most patients begin treatment between 25–35 years old.

The most commonly used primary substances are ketamine, methamphetamine, and GHB/GBL, with fewer patients using nitrous oxide and benzodiazepines.

60% of women are polysubstance users, with methamphetamine + GHB being the most common combination (37%).

All primary methamphetamine users struggle with dependence, with 37% identifying as transgender and 71% engaging in sex work. 37% of those who are dependent on methamphetamines had history of psychosis and been treated with antipsychotics.

66% of Ketamine users present with severe anxiety (GAD-7 score >15), and 56% experience ketamine bladder symptoms, requiring referral to Urology.

44% of women at the clinic have a diagnosis of PTSD, linked to trauma such as domestic violence, sexual abuse/assault, sex trafficking, and war-related trauma. These patients received therapy from the team psychologist or are referred to trauma-focused therapy within secondary mental health services.

**Conclusion:** This study identified ketamine and methamphetamine as the most commonly used primary psychoactive substances among female patients attending the Club Drugs Clinic. Methamphetamine dependence poses a significant risk for psychosis, while ketamine dependence increases the likelihood of developing ketamine-related bladder dysfunction, highlighting the importance of screening for cystitis symptoms. Additionally, the majority of patients reported a history of trauma and used substances as a coping mechanism. These findings emphasize the need for integrated care approaches, including close collaboration with trauma services, to enhance treatment outcomes and improve overall service provision for this vulnerable population.

